# Evidence for social cooperation in rodents by automated maze

**DOI:** 10.1038/srep29517

**Published:** 2016-07-05

**Authors:** Avi Avital, Shlomit Aga-Mizrachi, Salman Zubedat

**Affiliations:** 1 Behavioral Neuroscience Lab, Department of Physiology, Rappaport Faculty of Medicine, The Technion- Israel Institute of Technology, Haifa, and Emek Medical Center, Israel

## Abstract

Social cooperation is defined as a joint action for mutual benefit that depends on the individual and the counterparts’ behaviors. To gain valid evidence for social cooperation behavior we conducted a series of experiments in our suggested fully automated non-conditioned maze and depicted three major findings: (i) During 18 days of training the rats showed a progressive social learning curve as well as latent social learning; (ii) Examining the perceptual communication between the cooperating partners, we found a correlation between the available perceptual modalities and the social cooperation performance; and (iii) Investigating contextual learning as a competing process to the social cooperation, we found that additional contextual cues impaired the social cooperation performance. In conclusion, our suggested automated cooperation maze is designed to further our understanding of social cooperation under normal conditions, such as decision-making, and to examine the neural basis of social cooperation. A variety of neuropsychiatric disorders are characterized by disruptions in social behavior and social cognition, including depression, autism spectrum disorders, obsessive-compulsive disorder, and schizophrenia. Thus, on the pathological end, our maze for social cooperation evaluation can contribute significantly to the investigation of a wide range of social cooperation impairments in a rodent model.

Social cooperation is defined as a joint action for mutual benefit^1^ that depends not only on the individual behavior but also on the behaviors of others^2^. Cooperative behavior, a complex executive function, requires various social and cognitive skills from individuals which necessitates concurrent monitoring of ongoing social relationships^3^. Cooperative actions are essential to human social life. Engaging cooperative interaction requires that the cooperating partners are responsive to each other, oriented towards a mutual goal(s) and act in a supportive way to achieve the shared goal. Indeed, social cooperation in humans is evident in daily interactions, decision making, and cooperation toward mutual goals, that is modulated by positive^3^ or negative rewards^4^. Moreover, for cooperation to be favored, the individuals must gain utility either directly or indirectly^5^.

A variety of neuropsychiatric disorders are characterized by disruptions in social behavior and social cognition, including depression^6^,^7^, autism spectrum disorders^8^,^9^, bipolar disorder^10^, obsessive-compulsive disorder^11^ and schizophrenia^12^. In addition, Deweerdt^13^ has reported that withdrawing from social relationships can be a risk factor and an early sign for dementia.

A wide range of animal models for social cooperation has been suggested, including chimpanzees^14^, elephants^15^ and rooks^16^. Over the past two decades, several attempts to establish valid rodent models were proposed. Viana *et al*.^17^ cloned the prisoner’s dilemma game theory onto rats using a double T-maze. In summary, rats were trained to choose from cooperation or defection compartments that led to reward or punishment (e.g. payoff matrix). However, in addition to the social cooperation, this task involved a wide range of behavioral variables such as control of impulsivity and numerical discrimination.

Rutte and Taborsky^18^ introduced a method whereby rats were placed in a cage separated into two compartments by a wire mesh that required two rats learn to pull a stick fixed to a baited platform. Only one rat had access to the stick and the opportunity to move the baited platform into the cage, while access to the reward was always available only for its partner. However, this task put more emphasis on indirect- rather on direct-reciprocity. Further attempt to investigate cooperation in rats was carried out using operant chambers^19^. In this task, rats were individually trained for 4 weeks in a fixed ratio schedule of reinforcement for nose poke conditioning (light as the conditioned stimulus) that was rewarded if the nose poke was performed within 2 seconds. Thereafter, they were tested in pairs for mutual performance. However, the long period of individual training (28 days) may have affected the performance outcome of the pairs. Schuster and Berger^20^,^21^,^22^ introduced rats to a shuttle box, in which rats were first individually trained to run in order to receive a reward. In the next step, a pair of rats was required to coordinate their movement in order to receive mutual reward. However, this paradigm involved a series of operant conditioning-derived responses, due to the noise (conditioned stimulus) of the floor micro-switches operated each time the rats moved from one part of the chamber to the other (i.e each rat signaling the other to move forward). In a recent version of the same shuttle box^23^, in addition to the floor micro-switches, the researchers also used different lights to signal the coordinated movement through the different parts of the shuttle box. Thus, the floor micro-switches and the different lights may be considered as conditioned stimuli signaling the rats to move together. However, the studies by Schuster and Berger gave us the motivational ground to further explore social cooperation in a novel context. Thus, we aimed to form a task in which all partners gain equal reward, and are motivated to coordinate their behavior as a pair in a non-conditioned apparatus that is fully-automated and controlled by video tracking.

Our suggested social cooperation maze is comprised of a custom-made black lusterless perspex box divided vertically into two lanes. A successful trial of social cooperation involves a coordinated movement of two rats from the distal area of the maze toward the rewarding zone. Thereafter, peristaltic pumps are automatically activated to deliver mutual reward through liquid dispensers. Finally, in order to be rewarded again, rats are required to ‘restart’ their coordinative movement from the distal area of the maze toward the rewarding zone (detailed description in Fig. 1 and demonstration in video S1).

## Results

### Social cooperation: Learning and latent learning

Our aim is to develop a method that provides evidence for social cooperation in rodents via an automated maze. In the first step of developing such a method, a confirmation for an ongoing learning process was provided. The learning phase is comprised of 18 days in which an unchanging pair of rats cooperates toward the mutual reward. We found a significant elevation in the number of mutual rewards gained (F_(17,170)_ = 10.93, *P* < 0.0001, Fig. 2a). Starting on day 19, one of the partners in each pair was replaced by another trained partner (i.e. latent learning phase). Switching between different partners temporarily reduced the number of mutual rewards compared with day 18 (*P* < 0.002). However, a clear latent learning was observed, with higher number of rewards on day 19 as compared with the first day of learning. Measuring activity (Fig. 2b), we found a clear increase throughout the learning days (F_(17,357)_ = 6.90, *P* < 0.0001) that continued also during the latent learning phase. In order to examine the relationship between the aforementioned number of rewards and activity level, we calculated an efficacy index (Rewards^*^Activity^−1^). We found a significant and progressive efficacy curve (F_(17,357)_ = 13.87, *P* < 0.0001; Fig. 2c) similar to the pattern of the reward learning curve (Fig. 2a). In the latent learning phase we found a temporary reduction in the efficacy index compared with day 18 (*P* < 0.002). Yet, a clear latent learning was observed with higher efficacy index in day 19 compared with the first day of learning. Complementary to the activity measurement, we also examined the latency of the daily initial transition from zone A to C (Fig. 2d). Starting on day 8 and onwards, all latencies were significantly reduced (F_(23,483)_ = 8.96, *P* < 0.0001), compared to the first day of learning. Specifically, in both learning (days 8–18) and latent learning phases (days 19–24), the latencies reduced by 82.6% ± 3.94% and by 83.2% ± 2.75% (respectively), compared with the first day (*P* < 0.0001).

### The involvement of various perceptual modalities in social cooperation

Examining the perceptual communication between the cooperating partners, we evaluated the involvement of sensory modalities in the social cooperation performance. In humans as well as in some animals, the physical and the non- physical (i.e. verbal, visual, tactile, olfactory and auditory) communication are well developed. However, little is known about the non-physical communication in rodents. Thus, we aim to evaluate the possible differential contribution of various sensory modalities (i.e visual, tactile, olfactory and auditory) to the social cooperation ability. Moreover, to restrict possible communication to be only non-physical we utilized a divider between the cooperating rats. In order to distinguish between the sensory modalities, we tested cooperative abilities in four different divider conditions (Fig. S1).

A significant difference was found between the different divider conditions (F_(3,16)_ = 26.58, *P* < 0.0001). Post-hoc Tukey test revealed that the combined transparent & perforated divider led to the best performance (Fig. 3) as compared with the transparent, sealed or perforated dividers (*P* < 0.011; *P* < 0.0001; *P* < 0.0001; respectively). The transparent condition led to significantly better performance than sealed or perforated dividers (*P* < 0.003; *P* < 0.01; respectively), while no significant difference was found between the sealed and perforated dividers.

Thus, we found that the divider between the partners enabling all perceptual modalities yielded a superior social cooperation performance.

### The effects of contextual cues on social cooperation performance

Deciphering the effects of sensory modalities on social cooperation performance has led us to additionally examine whether the social cooperation performance also involves non-social cues (i.e. spatial cues that rats are known to be sensitive to). Thus, we examined the performance in the cooperation maze in a social versus social + contextual condition (Fig. S2). We found that both conditions led to significantly higher rewards rate, which manifested in a progressive learning curve (F_(17,442)_ = 28.79, *P* < 0.0001, Fig. 4a). Though the additional contextual cues could have subserved as a possible facilitator of the social cooperation performance, we found that the social + contextual group actually showed inferior performance compared with the social group (*P* < 0.05). No significant difference in activity was found between the groups (Fig. 4b). Similar to the reward learning curves, we found inferior efficacy index in the social + contextual group (F_(1,26)_ = 8.71, *P* < 0.007; Fig. 4c). Finally, no significant difference in the reduction of latencies was found between the groups (Fig. 4d).

Together, investigating contextual learning as a competing process to the social cooperation, we found that the additional contextual cues did not facilitate social cooperation performance, but rather impaired it.

### Sex differences

In order to examine possible sex differences in social cooperation ability, we compared the performance of female or male pairs. Both females and males progressively gained significant rate of rewards (Fig. 5a). However, starting on the first day of learning and onwards, the females performed better than the males (F(1,38) = 28.61, P < 0.0001). Likewise, the activity level of the females was significantly higher as compared with the males (F(1,38) = 40.36, P < 0.0001; Fig. 5b). Lastly, no significant differences between females and males were found in the efficacy index (Fig. 5c) or in the latency (Fig. 5d).

## Discussion

We first aimed to examine the ability of the rats to acquire social cooperation and to examine whether the social cooperation learning can also be latent - similar to other types of learning processes^24^. We found that during 18 days of social cooperation learning, the rats showed a progressive learning curve. Moreover, following the counterpart replacement, the social learning performance showed an expected decline, which was rapidly recovered. Additionally, the decline was significantly higher compared to the initial learning. These findings confirm that in our suggested automated social cooperation maze, indeed learning and latent social cooperation learning can be achieved, likened to other forms of learning^25^.

To further support our suggestion that social cooperation between the pair of rats is the ongoing process, we also examined the performance of an individual rat in the maze. Single rat performance led to a higher rate of rewards, activity level and efficacy index, compared with the cooperating pairs. Complementarily, the solitary rats group showed decreased latency of the transition from zone A to C. Indeed, one may consider individually shuttling from zone A to C as an easier task, since a solitary rat doesn’t need to coordinate with a counterpart in order to gain the reward.

Since we postulated that the communication between the partner’s functions as the most significant factor in the social cooperation performance, our second aim was to examine which sensory modality or combinations of sensory modalities orchestrated the perceptual ability of the rats to socially cooperate. We manipulated the divider between the rats in order to control the primary sensory modalities that mediate social cooperation, such as tactile, visual, auditory and olfactory sensory modalities. We found that enabling tactile perception led to poor performance while visual availability of the counterparts improved performance. Nonetheless, the divider that enabled perception of all sensory modalities yielded a superior social cooperation performance.

In contrast to social interaction that currently serves as the major model for social behavior^26^,^27^, in our maze no direct body contact was available, thus excluding behaviors such as biting, sniffing or grooming any part of the partner’s body. Furthermore, these physical contact behaviors are also suggested as aggression gestures and one can doubt the content validity when aiming to measure merely social behaviors^28^. As opposed to the subjective and manual scoring of the common social interaction behaviors, in our suggested social cooperation maze the social cooperation is scored automatically. Consequently, by focusing on the ability to socially cooperate toward mutual reward, our social cooperation maze is suggested as a valid method to evaluate sociability in a rat model.

Contextual learning is considered one of the leading cognitive processes in the rat’s natural navigation behavior. Thus, many rat models have utilized various context-dependant tasks such as the Morris water-maze, radial-maze, contextual fear-conditioning, etc^29^,^30^,^31^.

Accordingly, our third aim was to further verify that increasing social cooperation incidents are indeed attributed to social rather than other competing processes (i.e. non-social cues such as context). Therefore, we compared the performance in the social cooperation maze with or without contextual cues. Considering the important role of the contextual environment to the rat’s behavior, the additional contextual cues could have functioned as a possible facilitator of the social cooperation performance. However, we found an adverse effect of the additional contextual cues, likely due to an increased demand of attention to the supplemental contextual cues, in conjunction to the social cues, which may have led to an attention overload. Vigilance tasks that account for sustained attention, such as the social cooperation task, are resource demanding and thus comply with the theory of limited pool of information processing resources^32^. Information processing is a limited resource, and changes in the task are likely to cause qualitative changes in the final processing. Thus, adding contextual cues to the social information presumably led to adverse qualitative changes in the social cooperation performance that may be attributed to attention overload. To further support this postulation, we decreased the demand of attention to the social cues (i.e. single rats that run individually in the maze) and found that indeed under this low demand of attention condition, the single rats performed better than the cooperating pairs. Considering the contextual + social cues as a “high” demand of attention, the social cooperation as “intermediate”, and the single running as “low” demand of attention, we can see that the performance in term of reward and efficacy index are inversely correlated with this attention demand axis.

Our final aim was to examine sex differences in social cooperation performance. Previous human studies found equivocal sex differences in cooperation ability^33^. It has been suggested that females have higher cooperativeness, as supported by neuroanatomical findings^34^. We found that female rats are better in social cooperation than males. However, considering the efficacy index in our results, the increase in mutual rewards rate observed in female rats is positively correlated with the activity level. Thus, the superior performance of female rats is presumably derived from their higher level of activity.

For many human psychopathologies, the interactions between social and nonsocial deficits are poorly understood^35^, including in depression^36^, autism spectrum disorder^8^,^9^, bipolar disorder^37^,^38^, obsessive-compulsive disorder^39^, schizophrenia^38^,^40^ and psychopathy^41^,^42^.

Thus, our automated social cooperation maze presented here may better our understanding of the neurobiological underpinnings of social cooperation and the development of treatment strategies.

## Methods

### Animals

Male and female Wistar rats (weighing between 240–260 gr) were purchased from Harlan (Jerusalem, Israel) and were given seven days of acclimation in the institutional animal housing facility. Rats were housed three or four per cage (30_L_ × 30_W_ × 18_H_ cm, IVC System). Room temperature was maintained at 23 ± 1 °C with 67% humidity at 12:12 day/night cycle (lights on at 0700). Food and water access were allowed *ad-libitum*. This study was conducted in strict accordance with the recommendations of the Guide for the Care and Use of Laboratory Animals of the National Institutes of Health. The protocol was approved by the Technion’s (Israel Institute of Technology) Institutional Animal Care and Use Committee. All efforts were made to minimize animal suffering.

### Cooperative learning

**Figure 1:**
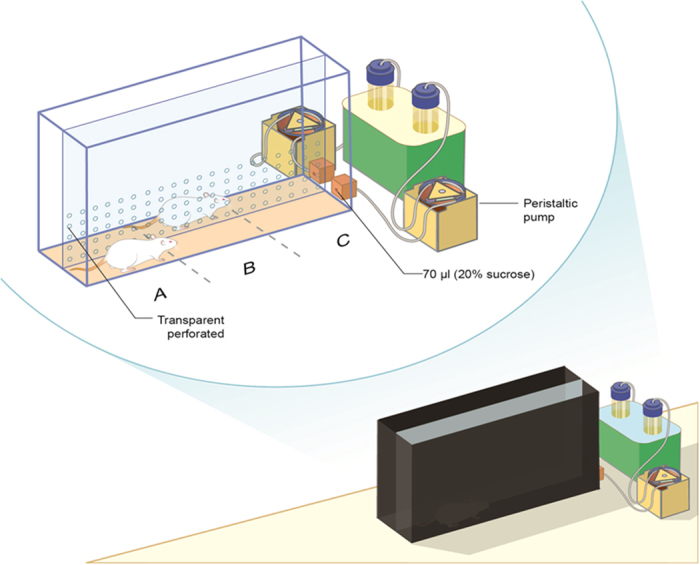
Social cooperation maze. Schematic illustration of the experimental objective. A black lusterless perspex box (40 cm W, 120 cm L, 50 cm H) divided vertically into two lanes by a transparent-perforated divider. The movement of the rats is monitored by a camera, providing the software with real-time input regarding the relative location of the rats in the 3 predefined zones (“A” to “B” to “C”). Upon the fulfillment of the computerized algorithm of cooperative condition, the peristaltic pumps provide mutual reward (70 μl, 20% sucrose drop) through the liquid dispensers. Re-gaining of mutual reward requires additional coordinated movement from zone “A” to “C”.

**Figure 2:**
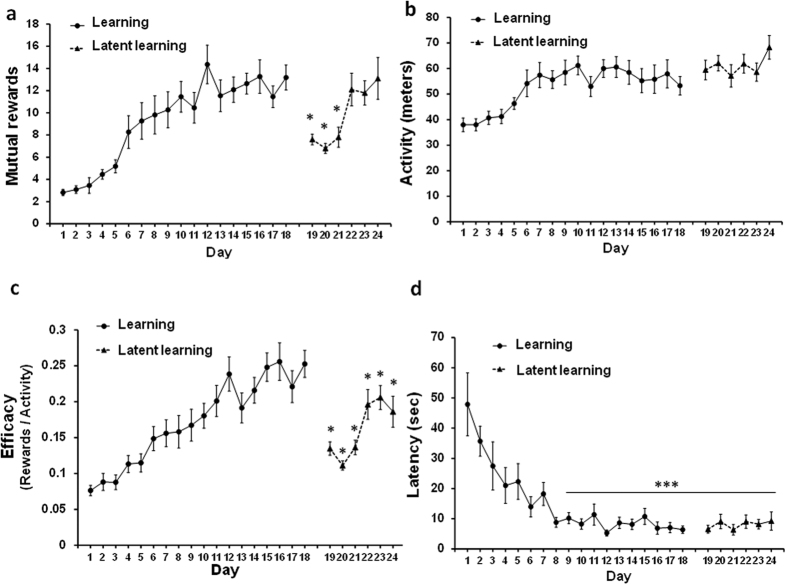
Social cooperation learning and latent learning. (**a**) During 18 days of learning, rats succeeded in gaining mutual rewards. On day 19 rats were assigned to another trained partner (coming from different home cages). The counterpart replacement led to a decrease in performance, yet higher than the first day of learning. This decrease rapidly recovered (days 19–24). (**b**) Gaining mutual rewards was accompanied by increased locomotor activity along all learning days. (**c**) To examine the utility of social cooperation, an efficacy index was calculated between the number of rewards and activity level. (**d**) The latency to the initial A-to-C mutual transition was evaluated. Error bars are SEM; n = 11 pairs of rats in each group (*P < 0.002; ***P < 0.0001).

**Figure 3:**
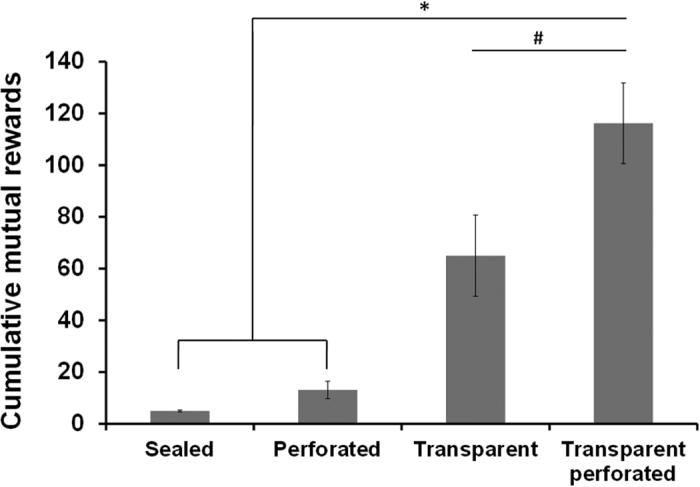
The involvement of various perceptual modalities in social cooperation. A comparison of possible differential contributions of various primary sensory modalities in mediating the social cooperation performance. Different dividers were placed between the cooperating rats in each experimental group, during 18 days of learning: (i) sealed (black lusterless perspex); (ii) perforated; (iii) transparent (transparent perspex) and (iv) transparent perforated. Error bars are SEM; n = 5 pairs in each group (^#^*P* < 0.011; *P < 0.0001).

**Figure 4:**
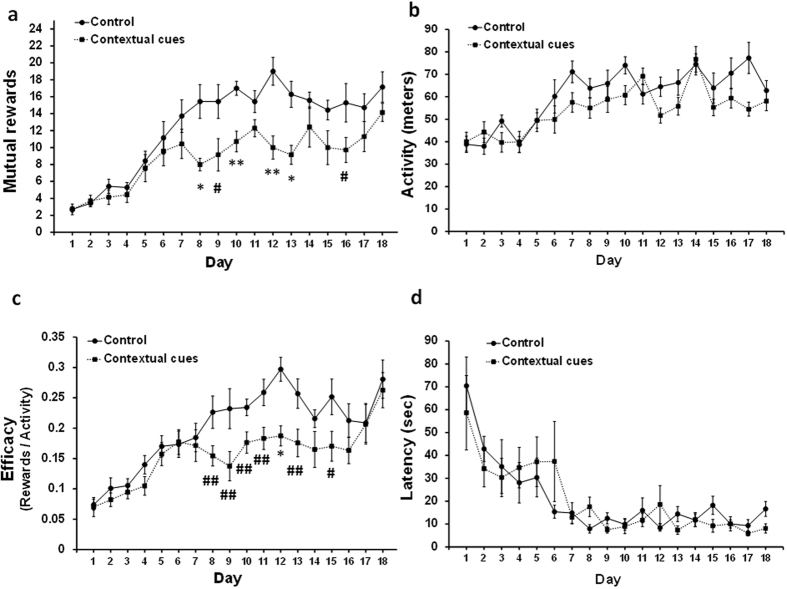
The effects of contextual cues on social cooperation performance. (**a**) The additional contextual cues impaired social cooperation performance, without altering (**b**) activity level. (**c**) The efficacy index of social cooperation was lower following the additional contextual cues. (**d**) The latency to the initial A-to-C mutual transition was not affected by the additional contextual cues. Error bars are SEM; n = 7 pairs of rats in each group (^#^*P* < 0.05; ^##^*P* < 0.025; *P < 0.001; **P < 0.0001).

**Figure 5:**
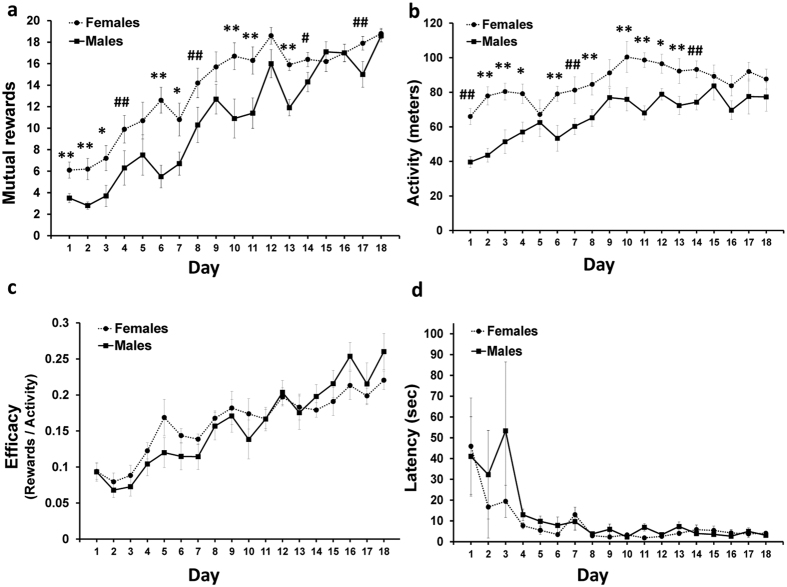
Sex differences. Female rats are better in social cooperation than males (**a**) and also showing higher level of activity (**b**). However, no significant differences were found in efficacy index (**c**) and latency to the initial A-to-C mutual transition (**d**). Error bars are SEM; n = 10 pairs of rats in each group (^#^*P* < 0.05; ^##^*P* < 0.025; *P < 0.001; **P < 0.0001).

The social cooperation learning was carried out in a dimly lit room (8lux) using a custom-made black lusterless perspex box (40 cm W, 120 cm L, 50 cm H) divided vertically into two lanes by transparent-perforated divider, allowing visual (illumination 5lux), auditory (background noise 58dB), tactile (the two lanes shared a continuous floor), and olfactory interactions between the two rats. A CC TV Panasonic camera placed ~2 m above the box, enabling a real time analysis of each rat’s movement (Fig. S3a) using Ethovision XT 7.0 software (Noldus, The Netherlands). The definition of the maze is based on the virtual 3 zones (Fig. 1) that perpendicularly divide the two lanes. When the two rats coordinate their movement throughout the virtual 3 zones (A to C), and the trial’s requirements are met by the algorithm programmed in the Ethovision software (Fig. S4), a TTL signal is automatically sent from the computer to the Noldus USB-IO box (Noldus, The Netherlands). The latter automatically activates the two peristaltic pumps (Campden Instruments LTD, England; modified by Noldus, The Netherlands; Fig. S3b). Each pump is connected to a single cup liquid receptacle (Med associates inc., Georgia, Florida, USA) with 18gauge pipe, located at the end of each lane, providing 70 μl 20% sucrose solution as reinforcement reward. While working, both pumps (placed ~0.5 m from the maze) add only 2dB to the background noise (58dB). The pumps are activated for 1.5 sec only after the rats accomplished the cooperation condition (i,e, arrived coordinately to zone C).

During the pre-learning phase, rats were habituated once individually to the cooperation maze for 10 min, and were allowed to consume sucrose at the end of the maze to overcome neophobia. Throughout the cooperative learning period, rats were water limited for 16–18 hours a day prior to the daily trial. The aim of limiting the availability of water was to enhance the motivation of the rats to perform the task. Previous reports indicated that the restricted availability of water for intervals of up to 24 hrs causes the sensation of thirst, but even chronic restriction schedules do not cause physiologic impairment^43^. Monitoring the body weight following the daily 16–18 hrs of water deprivation, we found that the rats’ body weight continued to normally increase (see additional Fig. S5). On the first day of learning and onward (for 18 consecutive days), each rat was placed in the maze with the same partner (coming from a different home cage) for 20 successive trials or 15 minutes maximum session duration. A trial was considered successful when the rats shuttled coordinately between the 3 predefined zones (“A” to “B” to “C”) within a maximal 10 sec delay from the adjacent zone and arrived to the end of the maze together (zone “C”). If this condition was fulfilled, then the peristaltic pumps were automatically activated and delivered a mutual sucrose reward (Video S1). A trial is restarted when both rats return to zone “A”. The rats do not receive a reward at the end of zone “C” if they were in different zones for more than 10 seconds or if they were more than one zone apart (“A” and “C”). The detailed algorithm is described in supplementary material (Fig. S4).

Finally, to further validate that our social cooperation maze is indeed measures cooperativeness, we examined the performance of solitary rats and found that they performed differently compared with the cooperating pairs (see Fig. S6).

### Latent social learning

**S**ubsequent to the acquisition of the cooperative learning, rats were assigned to another trained partner (coming from different home cages). In order to examine the latent social learning, we evaluated the effects of counterpart replacement on the learning performance for an additional six days (days 19–24), compared to their initial learning curves (days 1–18).

### Sensory modalities

In order to examine the involvement of the rats’ sensory modalities in mediating the social cooperation performance, we manipulated the divider between the cooperating rats in order to control the primary sensory modalities enabling communication between the cooperating partners, such as tactile, visual, auditory and olfactory. Accordingly, we utilized 4 versions of dividers: (i) sealed (black lusterless perspex); (ii) perforated; (iii) transparent (transparent perspex); and (iv) transparent perforated (Fig. S1). Rats were randomly allocated to each condition, and were evaluated for the total rewards after 18 days of social cooperation training.

### Contextual cues

In order to examine possible competing spatial effects to the social cooperation performance, we added different contextual cues on the lateral walls of the maze: “Zone A”- No cues, “Zone B”- Black and white vertical stripes, and “Zone C”- Red and White chessboard (Fig. S2). Rats were allocated to control (no contextual cues) or contextual cue groups, and their performance was evaluated during 18 days of social cooperation training.

### Statistical analysis

Data was analyzed for statistical significance using one-way analysis of variance (ANOVA) as well as ANOVA for repeated measures, followed by a Post-Hoc Tukey test or student’s *t*-test. When comparing between context conditions or sexes, we utilized two-way ANOVA for mixed-design, with context/sex as between-subject factor and days of learning as within-subject factor. A result was considered significant when *P* < 0.05. All tests were calculated as two-tailed using SPSS V21.0. Results are presented as means ± standard error of the means (SEM).

## Additional Information

**How to cite this article**: Avital, A. *et al*. Evidence for social cooperation in rodents by automated maze. *Sci. Rep*. **6**, 29517; doi: 10.1038/srep29517 (2016).

## Supplementary Material

Supplementary Information

Supplementary Movie S1
