# Supplementary material for: Evidence for social cooperation in rodents by automated maze
Source: Sci Rep. 2016 Jul 5;6:29517. doi: 10.1038/srep29517 (PMC4932492; doi:10.1038/srep29517)
Supplement: Supplementary Information [file srep29517-s1.pdf]

Evidence for social cooperation in rodents by automated maze

Avi Avital*, Shlomit Aga-Mizrachi and Salman Zubedat

Supplemental information

Supplementary Video 1. A representative video of social cooperation performance on day 18 of learning.

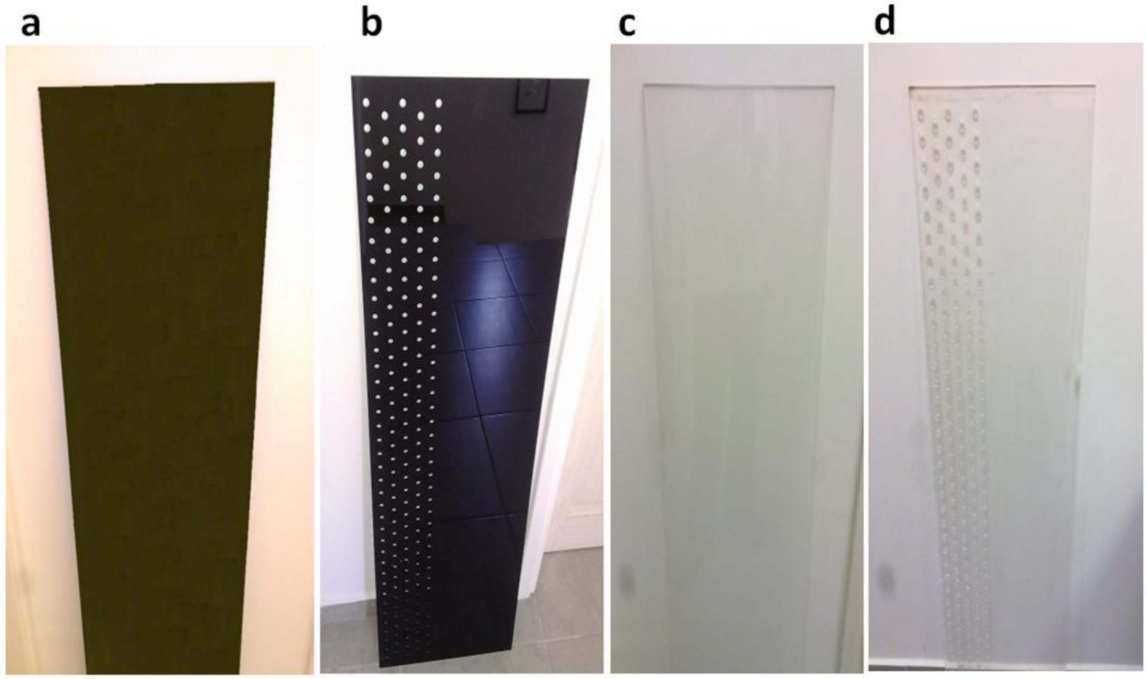

Supplementary Figure 1. The involvement of various sensory modalities in social cooperation, utilizing different dividers: (a) sealed (black lusterless perspex); (b) perforated; (c) transparent (transparent perspex) and (s) transparent perforated.

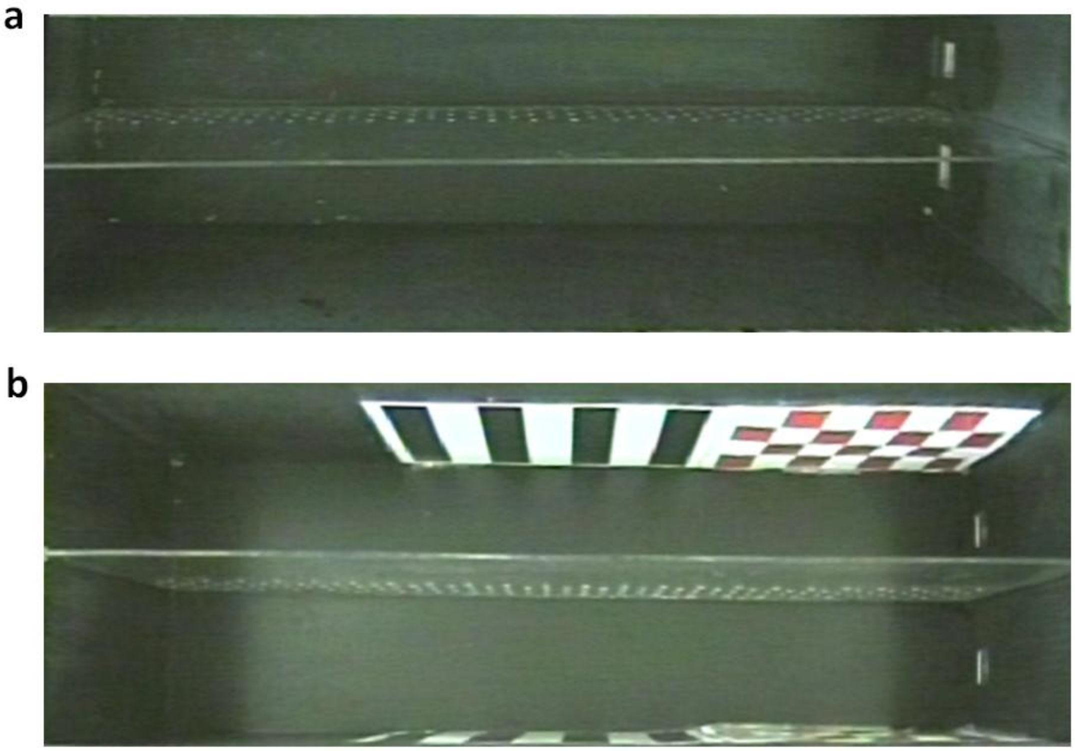

Supplementary Figure 2. Social cooperation maze (a) without any contextual cues or (b) with different contextual cues on the lateral walls of the maze.

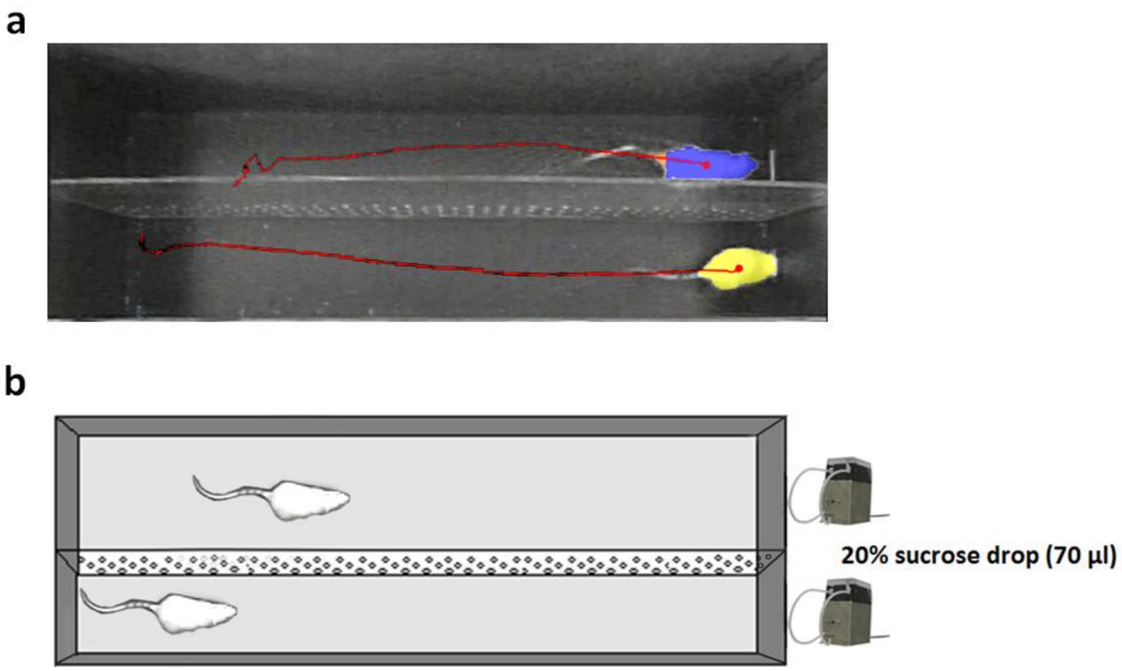

Supplementary Figure 3. (a) A real-time computerized tracking analysis of each rat's movement, enabling the activation of (b) the computer-controlled peristaltic pumps providing reinforcement reward.

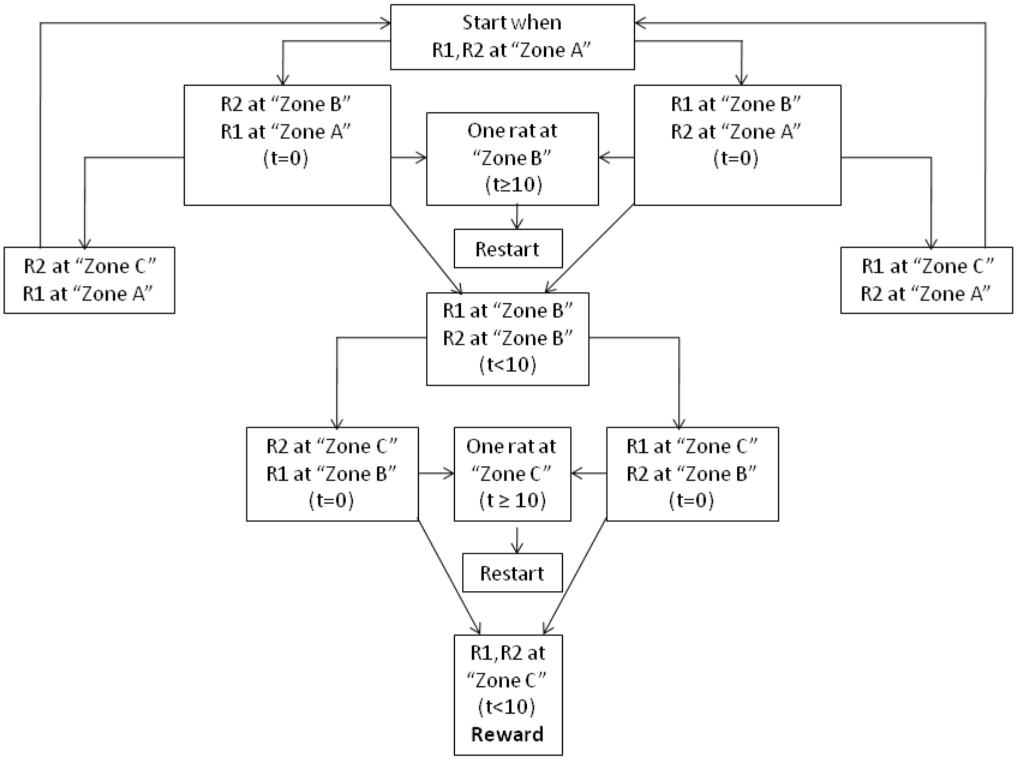

Supplementary Figure 4. The algorithm implemented in the computerized on-line analysis of social cooperation.

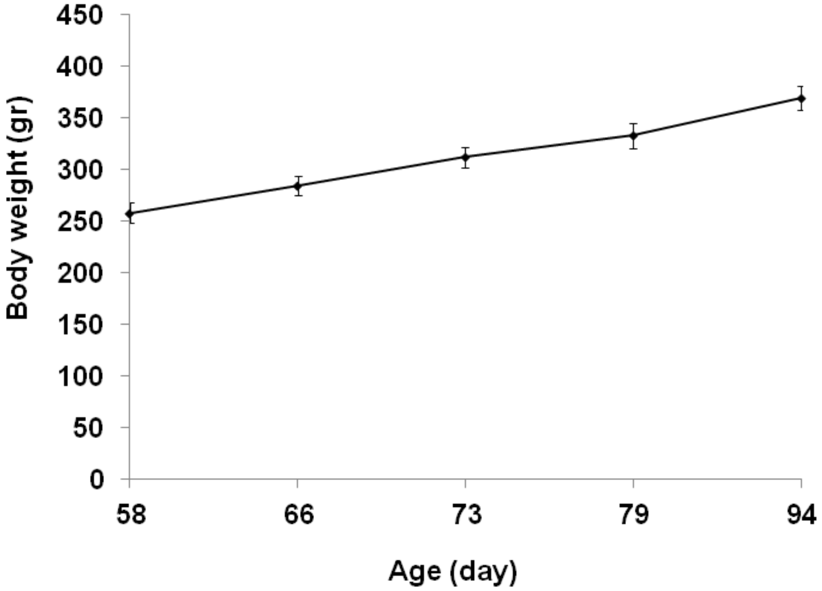

Supplementary Figure 5. Rats body weight during social cooperation period that included daily 16-18 hrs of water deprivation.

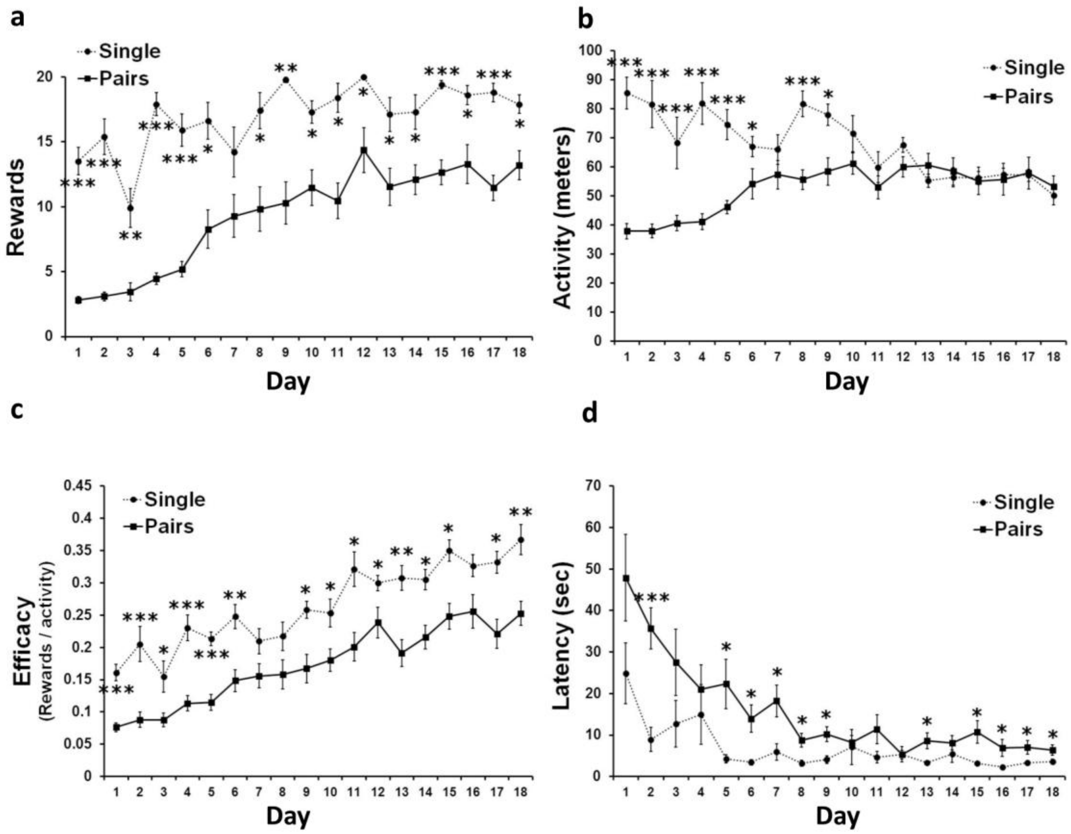

Supplementary Figure 6. Single rats ran individually in the same maze as the cooperating pairs. (a) Compared with the cooperating pairs, single rat running led to higher rate of reward during 18 days of learning. (b) The activity level of the single rats was higher during the first 9 days and then was similar to the activity level of the cooperating pairs. (c) Compared with the cooperating pairs, the efficacy index of single rats was higher during 18 days of learning. (d) The latency to the initial A-to-C solitary transition was lower compared with the cooperating pairs. Error bars are SEM; n=10 single rats, n=11 cooperating pairs (**P*<0.05; ** *P*<0.001; ****P*<0.0001).
